# PhenoGO: an integrated resource for the multiscale mining of clinical and biological data

**DOI:** 10.1186/1471-2105-10-S2-S8

**Published:** 2009-02-05

**Authors:** Lee T Sam, Eneida A Mendonça, Jianrong Li, Judith Blake, Carol Friedman, Yves A Lussier

**Affiliations:** 1 Center for Biomedical Informatics, Department of Medicine, The University of Chicago, Chicago, IL, USA; 2 Department of Biomedical Informatics, Columbia University, New York, NY, USA; 3 The Jackson Laboratory, Bar Harbor, ME, USA; 4 The University of Michigan, Ann Arbor, MI, USA

## Abstract

The evolving complexity of genome-scale experiments has increasingly centralized the role of a highly computable, accurate, and comprehensive resource spanning multiple biological scales and viewpoints. To provide a resource to meet this need, we have significantly extended the PhenoGO database with gene-disease specific annotations and included an additional ten species. This a computationally-derived resource is primarily intended to provide phenotypic context (cell type, tissue, organ, and disease) for mining existing associations between gene products and GO terms specified in the Gene Ontology Databases Automated natural language processing (BioMedLEE) and computational ontology (PhenOS) methods were used to derive these relationships from the literature, expanding the database with information from ten additional species to include over 600,000 phenotypic contexts spanning eleven species from five GO annotation databases. A comprehensive evaluation evaluating the mappings (*n *= 300) found precision (positive predictive value) at 85%, and recall (sensitivity) at 76%. Phenotypes are encoded in general purpose ontologies such as Cell Ontology, the Unified Medical Language System, and in specialized ontologies such as the Mouse Anatomy and the Mammalian Phenotype Ontology. A web portal has also been developed, allowing for advanced filtering and querying of the database as well as download of the entire dataset .

## Introduction and significance

The advent of high throughput techniques in the biological realm and the concomitant exponential increase in the amount of computing power offered has made an unprecedented amount of biological data available for complex analysis not possible in the past. Studies of the proteomes of entire organisms have now been made possible, facilitating analyses never before possible. This has been particularly notable in the study of complex diseases. The addition of diseases and disorders to the phenotypic annotations as part of the expansion and extension effort has made the database a prime resource for multi-scale systems analyses of biological significance across a large number species. For example, a number of studies have sought to amalgamate the human proteome with known diseases and their associated genes and protein products. PhenoGO was applied to one of the first of such studies, aimed at elucidating the molecular mechanisms underlying complex diseases *en masse *[1]. Similar studies have applied text mining strategies over clinical data sources such as the Online Mendelian Inheritance in Man with varying degrees of success [2-5].

The Gene Ontology was established to provide a comprehensive, universal resource with which to characterize molecular elements in terms of their characterized traits and functions. However, the functional concepts often attributed to genes only exist within some phenotypic context – which is almost as equally often left out. PhenoGO is a multi-organism database that provides phenotypic context to existing associations between gene products and GO terms as specified in the *Gene Ontology Annotations *(**GOA**) [6]. Context for identifiers are mapped to widely employed biological ontologies, including the *Cell Type Ontol*ogy (**CO**) [7], the *Unified Medical Language System *(**UMLS**) [8], and National Library of Medicine's *Medical Subject Headings *terminology (**MeSH**) [9] and some specialized ontologies such as the Mammalian Phenotype Ontology (**MP**) [10] and adult Mouse Anatomy (**MA**) [11]. This set of ontologies and terminologies allows for the contextualization at multiple scales of biology; mutations in a gene can be analyzed from multiple perspectives, from the resulting disruption of a biological process, and subsequent dysfunction in a cellular context, to changes in anatomy and morphology, and scaling up to the manifest disorder on an organismal level.

The original release of the PhenoGO database was focused on mouse phenotypes. The database now includes annotations for eleven of the species defined in the *National Center for Biotechnology Information *(**NCBI**) taxonomy [12], including *Schizosaccharomyces pombe*, *Saccharomyces cerevisiae*, *Caenorhabditis elegans*, *Drosophila melanogaster*, *Drosophila sp.*, *Danio rerio*, *Gallus gallus*, *Homo sapiens*, *Bos taurus*, *Mus musculus*, and *Rattus norvegicus*. Data sources include GO annotations from the Saccharomyces Genome Database (SGD) [13], Wormbase [14], Flybase [15], the Zebrafish Information Network (ZFIN) [16], the European Bioinformatics Institute (EBI), Mouse Genome Informatics at the Jackson Laboratories (MGI) [17], and the Rat Genome Database (RGD) [18]. The integration of knowledge from these heterogeneous sources using established, standardized coding schemes enables broader application of multiscale systems approaches to the analysis of complex disease and biological processes. As the PhenoGO dataset was developed to facilitate high throughput mining of experimental, phenotypic or disease contexts associated to gene-to-GO annotations, the expansion of the database was focused primarily on species that are established model systems.

## Background

### Function and phenotypic context

The Gene Ontology is a one of the most widely used resources for the functional characterization of biological entities. The ontology is used by virtually every database referencing proteins and genes, and numerous systems rely on it for the codification and prediction of function [19-21]. The concept of function has a complicated relationship to the cellular and phenotypic context due to a number of factors such as regulatory characteristics, alternative splicing, epigenetic effects, and other post-translational modifications. Even alone, the concept of function encompasses numerous genomic, genetic, and molecular features spanning multiple scales of biology [22]. These features include protein interaction partners, biological pathway membership, genomic context and position, tissue type, and cellular localization, all of which affect the role of a biological entity's function in varying environmental and temporal contexts. This gives rise to cases where phenotypic information is necessary to resolve conflicting or inconsistent functional annotations.

### Phenotypic annotations of genes

A number of model organism databases (e.g. Mouse Genome Database, Flybase, etc.) provide phenotypic contexts associated to a gene, but this context is not transferred to the GO terms also associated to the gene. As illustrated in the following example, phenotypic contexts are not fully transitive to GO annotations related to a common gene; in other words, every phenotypic context associated to a gene does not necessarily apply to every GO term also associated to the gene.

The well known athymic "nude mouse" *Foxn1*^*nu*^/*Foxn1*^*nu*^, which is widely used in graft and cancer research, can serve as a proof of concept of the value of phenotypic cellular contexts for GO annotations. Among others, the following GO annotations are provided in the Mouse Genome Database with the Forkhead box N1 (Winged-helix transcription factor nude) gene [MGI:102949 *Foxn1*]: "keratinocyte differentiation [GO:003216]". Now, a single genetic mutation is responsible for the disruption of the winged helix protein from the region of Chromosome 11 of the *Foxn1*^*nu*^/*Foxn1*^*nu *^mutant mouse, and most importantly, this mutated protein is expressed in the skin [23], and in other anatomies, such as the thymus, ovaries, etc.. Therefore, GO annotations of *Foxn1*^*nu *^allele could be refined with the anatomical context. In this case, the "keratinocyte differentiation" is specific to the skin context, rather than the context of the thymus or ovaries.

### Automated mapping of phenotypes

To our knowledge, there are no automated methods for the mapping of phenotypes to GO annotations. The natural language processing (*NLP*) component of PhenoGO utilizes an existing system, called BioMedLEE, which is under development jointly by the Friedman and Lussier research groups [24]. The BioMedLEE system is an adaptation of the MedLEE system, which accurately extracts and encodes clinical phenotypic information in patient reports [25]. BioMedLEE extracts and encodes genotype-phenotype relations from information in text. Chen and colleagues described a previous version of BioMedLEE that extracted phenotypic information, but did not map textual terms to codes as the current system does [25].

### Computational ontologies

The Phenotype Organizer System (***PhenOS***) is a system developed by the Lussier Research Group with the purpose of bridging the gaps among heterogeneous biomedical terminologies. The system provides lexico-semantic and model-theoretic methods for automatically mapping one ontology to another independently of the UMLS, and organizing and structuring phenotypes across heterogeneous datasets [26,27]. Specific methods of PhenOS were used in the current study to integrate phenomic knowledge structures via structured terminologies [28].

## Database contents

**Figure 1:**
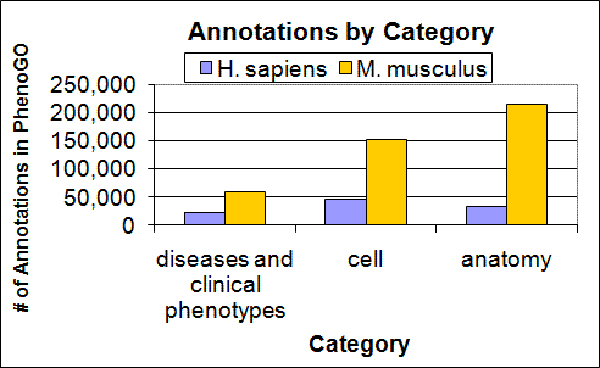
Annotations in PhenoGO by category in Human and Mouse.

The PhenoGO database contains phenotypic annotation for gene-GO relationships in eleven species, expanded significantly from its first iteration focusing on the mouse. The database currently contains over half a million unique annotations, derived using both natural language processing and computational terminology techniques (outlined in the Methods section). Table 1 shows the distribution of annotations across the eleven species represented in the database. The distribution of annotations according to phenotypic context code is shown in Figure 1.

**Table 1:** Annotations in the PhenoGO database, stratified by species

**Taxon**	**Name**	**# Annotations**
4896	*Schizosaccharomyces pombe*	344
4932	*Saccharomyces cerevisiae*	4,192
6239	*Caenorhabditis elegans*	12,212
7227	*Drosophila melanogaster*	91,782
7242	*Drosophila sp.*	238
7955	*Danio rerio*	3,142
9031	*Gallus gallus*	358
9606	*Homo sapiens*	102,262
9913	*Bos taurus*	804
10090	*Mus musculus*	427,275
10116	*Rattus norvegicus*	15,432
**Total**		658,041

The PhenoGO database is made publicly accessible through a web portal using Java Server Pages to access an underlying mySQL database at .

**Figure 2:**
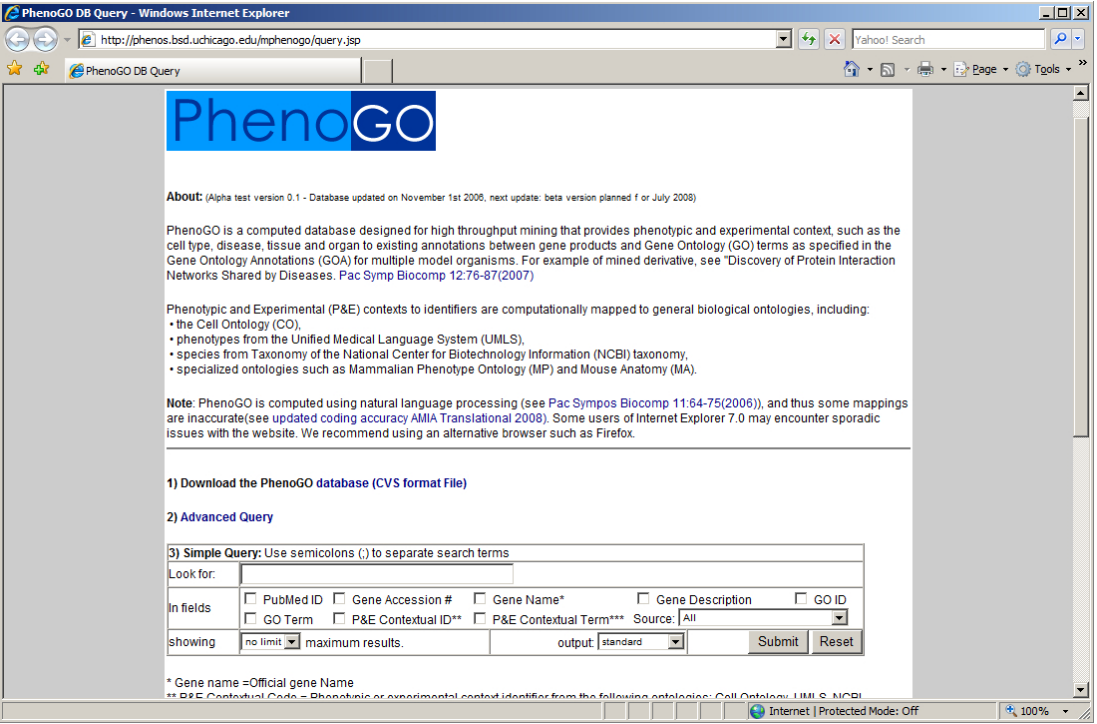
**The PhenoGO Portal and basic query**. The basic query interface was designed to be inclusive in gathering results, returning annotations in the database matching any one or more of the user's query terms.

**Figure 3:**
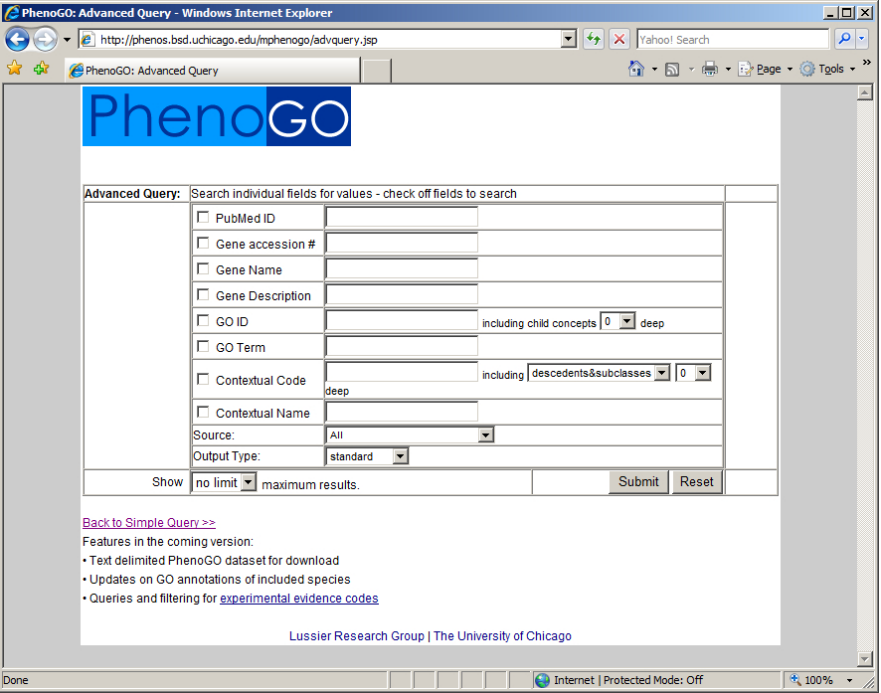
**The PhenoGO Advanced Query**. The advanced query page allows users to quickly narrow down their results of interest, allowing for hierarchical queries of the database using GO and Phenotypic and Experimental contextual queries of interest.

**Figure 4:**
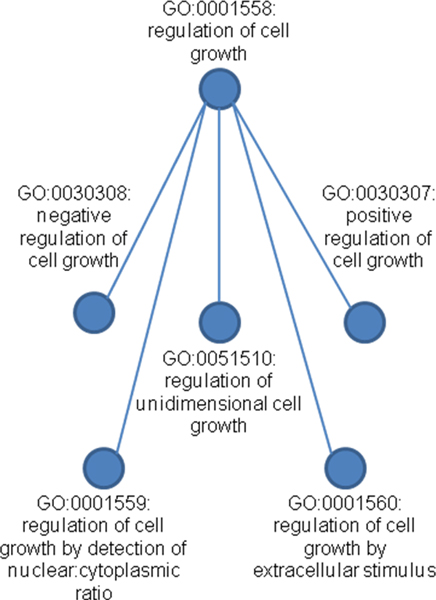
**A hierarchal query in the Gene Ontology will return results from descendent concepts**. The hierarchical query of GO:0001558 (regulation of cell growth) will result in the retrieval of annotations associated with several descendent concepts in the Gene Ontology.

The web portal accommodates simple queries designed to retrieve as much information as possible and complex queries aimed at retrieving specific slices of data. The basic query interface, shown in Figure 2, allows for retrieval according to PubMed ID, gene accession number, gene name, gene description, GO code, GO name, contextual phenotype name, contextual phenotype code, and species. An advanced query interface allows for the recall of entire hierarchies of ontologically associated entries based on GO and phenotypic context codes, as shown in Figure 3. For example, a hierarchical search for GO:0001558 (regulation of cell growth) will also search for annotations related to GO:0030308 (negative regulation of cell growth), GO:0030307 (positive regulation of cell growth), GO:0001559 (regulation of cell growth by detection of nuclear:cytoplasmic ratio), GO:0001560 (regulation of cell growth by extracellular stimulus), and GO:0051510 (regulation of unidimensional cell growth). This is shown in Figure 4.

Resulting data is available in the form of formatted HTML or tab-delimited text output for computational use. This interface exposes the entirety of the database for export into a computational study unlike many other resources, which lack support for large-scale data export. Furthermore, download of the entire dataset as a single file is available from the website.

## Methods

The addition of ten additional species to the database was done using the existing PhenoGO data extraction pipeline. Gene Ontology annotations for *Schizosaccharomyces pombe*, *Saccharomyces cerevisiae*, *Caenorhabditis elegans*, *Drosophila melanogaster*, *Danio rerio*, *Gallus gallus*, *Homo sapiens*, *Bos taurus*, *Mus musculus*, and *Rattus norvegicus *were downloaded from the current annotations section of the Gene Ontology website at . Phenotypic associations were made using a combination of methods utilizing natural language processing and computational terminology approaches. The natural language processing approach applied the BioMedLEE NLP engine [25] to derive annotated lists of genes, their related GO terms, and phenotypic associations given a list of PubMed abstracts. Additional mappings are derived using the existing MeSH annotations found in abstracts. The resultant output was then processed with the PhenOS system, yielding the final gene-GO-phenotype entries. The method is described in detail in [29].

Diseases were annotated through the extension and expansion of the original processing pipeline designed for the annotation of cellular and anatomical contexts. First, the two paths of the encoding pipeline were modified to handle disease and clinical finding associated phenotypic context. Disease and clinical finding-related semantic types from the UMLS were introduced into the BioMedLEE knowledge base to supplement the NLP-driven encoding of disease phenotypes while disease associated MeSH headings were added into the system to enable direct extraction of these annotations. To ensure consistency, disease and clinical finding-associated MeSH headings and UMLS terms were chosen using the same semantic type filtering rules. Additionally, grammar rules specific for the recognition of diseases and clinical findings from the MedLEE system were also added to the BioMedLEE ruleset to enable the encoding of the new class of contexts [24].

The gene accession number-GO code-phenotype entries resulting from this pipeline are enriched with full-text annotations for terms and names to enhance data readability and searchability using a series of Perl scripts which match gene accession numbers and GO identifiers to their names and descriptions. Data correlating identifier codes and accession numbers are taken from the Gene Ontology description files and the gene description files from UniGene, UniProt, MGI, RGD, SGD, Wormbase, and Flybase.

A web portal was developed to provide access and filtering functionality for the database. This portal provides two modes of querying the data. The first is a simple query which users are first exposed to on the front page of the portal. It allows for a search by all the fields of the database, including Pubmed ID, gene accession number, gene name, gene description, GO ID code, GO Term name, phenotype or experimental context code, and phenotype or experimental context description. This query mechanism is designed to provide users with a large number of results from the database, essentially corresponding to a logical OR query for all the query terms. An advanced query system is also made available to provide more exact results. The advanced query allows for searches based on the same fields as the basic interface, however it is focused on providing sets of results passing a number of strict criteria. This equates to a logical AND query between all the search terms specified by the user in specific fields. The interface also makes use of the structured organization of the Gene Ontology, the UMLS, and the Cell Ontology to provide hierarchical query functionality for the GO and context fields. This is done through the generation of a number of ancestor-descendent tables which are recursively processed at query time to determine all descendents or descendents and subclasses of user-specified contextual or GO terms.

The comprehensive evaluation was completed independently by two reviewers, each of whom reviewed 300 entries from the human and mouse subsets of the database. These entries were randomly retrieved directly from the PhenoGO mySQL database in 100 entry sets and stratified by context type. These four context types were defined by the BioMedLEE NLP engine; 'cell' involving annotations pertaining to cells and cell types, 'anatomy' encompassing annotations related to anatomies and morphologies, and 'problem' and 'problemdescr' describing diseases and disorders. The context types 'problem' and 'problemdescr' were merged into a general class encompassing both diseases and clinical phenotypes due to their similarity. Evaluation of this class was achieved using 50 random entries examined by two reviewers independently. Confidence intervals are calculated using the confidence level for proportions equation.

$$\hat{p}\pm \sqrt{\frac{\hat{p}(1-\hat{p})}{N}}$$

Our evaluation metrics were structured such that a true positive is only scored when the pipeline is able to both accurately encode a phenotype and associate it to its corresponding Gene-GO pair. Precision was measured by manually evaluating the entries recalled from the random draw and determining the percentage of correct annotations out of the total drawn entries. Recall was evaluated by randomly drawing encoded sentences from the NLP evaluated literature and computing the fraction which were seen in the encoded dataset.

## Results and discussion

**Figure 5:**
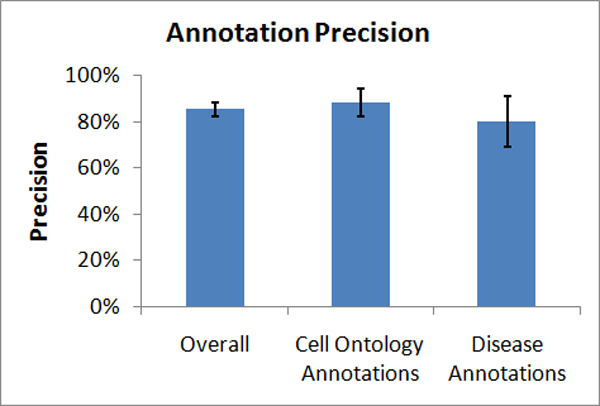
Precision in each of three evaluations n = 300.

As shown in Figure 5, a comprehensive evaluation of a random set of 300 phenotypic annotations was conducted to measure the accuracies of the mappings after the initial expansion of the database, adding many more organisms and the disease class: precision (positive predictive value) was measured at 85% (95% Confidence Interval: 82%–89%), and recall (sensitivity) was measured at 76% (95% CI: 69–83%). An additional 92,910 annotations were added after the comprehensive evaluation was complete. Particular attention was also focused on the newly added disease focused annotations in humans, where an evaluation done over 50 random annotations measured precision at 80% (95% CI: 69%–90%). Also of interest are the 115,464 phenotypic contexts of the CO mapped to GO annotations with a precision of 88% (95% CI: 82%–94%) and a recall of 79% (95% CI: 69%–89%). Table 1 illustrates the distribution of phenotypic annotation in the database.

The high levels of accuracy demonstrated in the evaluation show that the PhenoGO resource can be used as a data source in a number of computational applications. This high level of accuracy was valuable in conducting our study of human diseases and their relationships through the creation of a phenome-interactome network. This network was composed of the gene-disease relationships found in PhenoGO coupled with molecular interactions in a high-quality, human curated protein-protein interaction network [1]. Similar studies by Lage et al. [2] and Goh et al. [30] used similar phenotype data derived though natural language processing of OMIM. The popularity of OMIM reveals both the high quality of its contents and the relative paucity of readily computable genome-phenome resources available to researchers.

This is particularly important for candidate gene prioritization applications where the availability of accurate, precise, and computable knowledge is a necessity in order to train classifiers or filter biological candidates. In addition, the application of context in many studies should help further pare down the candidate list based on temporal expression patterns and localization. Alternatively, using cell ontology, which is a new authoritative organization of cell types, one can use PhenoGO and GO annotations databases to create a high throughput comparative analysis of gene-GO annotations across species. Similarly, many other automated predictive or analytic systems can be built over the PhenoGO phenotypic contexts related to specific GO annotations.

## Limitations

The current alpha version of the  database does not provide a query over the specific taxon or the "experimental evidence codes" found in Gene Ontology. An update of the dataset content is conducted annually in July.

## Conclusion and future work

This paper demonstrates the PhenoGO resource, a multi-organism database augmenting existing Gene Ontology annotations with phenotypic context using a number of widely used structured ontologies. An evaluation of the contextual modifications demonstrates that the resource reaches a high level of accuracy, comparable to other existing biological resources. By enriching existing functional annotations with phenotypic context, we increase the specificity and computability of the annotations. The expansion of the database to include ten additional species and addition of disease annotations makes it a prime resource for high-throughput experiments examining the complexities underlying disease and their associated biological processes. Our objective is to provide an accurate and regularly updated open source database of phenotypic and contextual annotations for high throughput access and analysis by the biological and bioinformatics communities, accessible in a structured, readily computable form. As a consequence, we intend to revise the automated BioMedLEE and PhenOS technologies to increase the recall and precision of the system by providing methods for filtering by levels of predicted accuracy. Additionally, during the coming update in July, additional advanced query capabilities will be added across species and "experimental evidence codes" found in GO.

## Competing interests

The authors declare that they have no competing interests.

## Authors' contributions

Lussier was responsible of the overall design and contributed to the R&D of BioMedLEE and PhenoGO. Friedman contributed significantly to BioMedLEE. Sam and Li were involved in the implementation and updates respectively. Sam, Mendonca, Blake, Friedman and Lussier contributed to the evaluation. Sam and Lussier were involved in the discussion.
